# A DFT study on hydrogen diffusion across zinc surfaces at low coverage

**DOI:** 10.1038/s41598-025-15064-y

**Published:** 2025-08-13

**Authors:** Mihaela Buga, Teodora Murariu, Larisa-Milena Pioraş-Ţimbolmaş, Carmen Tripon, Luiza Buimaga-Iarinca, Cristian Morari

**Affiliations:** 1 National Research and Development Institute for Cryogenic and Isotopic Technologies Rm.Valcea ICSI ENERGY, 4 Uzinei Str., 240050 Rm.Valcea, Romania; 2 National Institute for Research and Development of Isotopic and Molecular Technologies, 67-103 Donat, 400293 Cluj Napoca, Romania; 3 Babeş-Bolyai University, Faculty of Physics, 1 Mihail Kogălniceanu, 400347 Cluj-Napoca, Romania

**Keywords:** Hydrogen diffusion, Zinc surface, DFT, Van der Waals, Molecular dynamics, Chemistry, Materials science, Physics

## Abstract

The mechanism governing hydrogen diffusion on Zn surfaces plays a crucial role in the hydrogen evolution reaction (HER). This reaction is vital for various technological applications, particularly its influence on the cycling stability of aqueous Zn-ion batteries. However, the fundamental relationship between hydrogen diffusion, Zn surface properties, and HER remains unclear. In this study, we investigate the dynamics of hydrogen on Zn surfaces using density functional theory and ab initio molecular dynamics. Molecular dynamics data allow us to estimate the vibrational density of states of adsorbed hydrogen and to calculate the diffusion coefficients. Vibrational data indicate that the vibrational motion parallel to the surface of the hydrogen atom has a similar frequency to that of the surface phonons, suggesting the role of vibrations in the diffusion process; moreover, we calculate the diffusion coefficient on top of a frozen surface and get values that are several orders of magnitude lower than those obtained in the presence of surface phonons, i.e. $\approx 10^{-8}$ ${\text{m}}^2/{\text{s}}$.

## Introduction

Diffusion of hydrogen on metal surfaces is essential for a whole range of technological applications, mainly in the field of energy storage. Among these, zinc–air batteries have attracted significant attention as promising candidates for energy storage applications due to their high energy density, low cost, and environmentally friendly characteristics^1–3^. Central to the performance of zinc–air batteries is the hydrogen evolution reaction (HER) occurring on the zinc anode surface. During discharging, the zinc anode undergoes oxidation, releasing electrons, which are then used to reduce oxygen at the cathode. However, during charging or at high overpotentials, the reverse HER can take place on the anode, leading to the evolution of hydrogen gas. This side reaction can significantly hinder the efficiency and overall performance of zinc–air batteries, limiting their commercial viability and capacity retention^4–13^.

Understanding the mechanisms of HER on zinc anode surfaces is critical for optimizing the performance of zinc–air batteries. The HER involves several steps, including the adsorption of hydrogen atoms on the zinc surface, their subsequent diffusion across the surface, and the formation of hydrogen gas through electrochemical reactions^14–16^. Factors such as the crystallographic structure of the zinc anode, the presence of surface defects, and the electrochemical environment all play crucial roles in determining the efficiency of the hydrogen evolution process^17,18^. Additionally, the interaction between the evolving hydrogen and the zinc surface can lead to issues such as passivation, corrosion, and the formation of unwanted by-products, which further complicate the management of HER in zinc–air batteries^19–21^.

This study aims to provide a comprehensive understanding of hydrogen diffusion on zinc surfaces in the low-coverage regime, where the probability of hydrogen-hydrogen collisions on the surface is minimal, ensuring that atomic jumps between adsorption sites remain unhindered.

By exploring the fundamental processes of hydrogen adsorption and diffusion, we pursue two main objectives. First, we seek to develop an accurate model for hydrogen diffusion on zinc surfaces under low coverage conditions and compare it to other DFT results of hydrogen-metallic surface interaction^22–24^. Second, we aim to assess the reliability of theoretical approaches by comparing them to experimental data, paving the way for more complex simulations that can provide an explicit description of the hydrogen evolution reaction (HER). Ultimately, enhancing our understanding of hydrogen diffusion at the zinc anode will contribute to the development of more efficient, durable, and commercially viable zinc–air energy storage systems.

Recent studies^1,25^ have highlighted the necessity of maintaining an atomically flat Zn anode surface to minimize HER activity. This conclusion was drawn from DFT analyses of HER on various Zn surface orientations (e.g., Zn(002), (100), (101), (102), and (103)), linking HER activity to variations in the generalized coordination number on non-ideal surfaces^1^.

However, the models presented in^1^ rely on DFT calculations at specific static configurations, neglecting the influence of dynamics and temperature. In particular, substrate phonons are expected to play a crucial role in the diffusion process, as the deformation of the surface due to vibrational modes is directly related to hydrogen atom jumps between equilibrium sites. Our goal is to provide a dynamical characterization of hydrogen diffusion using a combination of DFT and molecular dynamics simulations. This approach will allow us to examine the impact of surface phonons on diffusion, as well as the effect of temperature, which is inherently linked to the vibrational motion of the substrate.

## Methods

### DFT computational setup

The simulations were carried out in the frame of density functional theory (DFT) using the Siesta code^26^ (see details below). We used the BH corrected exchange correlation functional which has proven to work well for molecule surface phenomena^27–29^. The LCAO basis set used was of double-zeta polarized basis type with an energy shift of 30 meV.

For the Zn bulk, we start with a hexagonal close-packed structure (*hcp*, see the data at^30^) with two atoms/cell and the cell parameters $a=b=2.6649$ Å and $c = 4.9468$ Å. The cell and atomic positions for the bulk structure were relaxed by DFT up to a gradient of 0.01 eV/Å using a $10 \times 10 \times 10$ points mesh in the reciprocal space; the final structure retains the *hcp* symmetry with $a=b=2.6606$ Å and $c=4.9444$ Å . The slab model was built form periodically repeated cells of this structure (i.e. a total of $4 \times 5 \times 3$ unit cells along *a*, *b*, *c* cell parameters of the *hcp* structure) for a total of 120 Zn atoms. We allowed a 15 Å vacuum gap between layers to simulate the surface effects.

After the structural relaxation, a molecular dynamics simulation was conducted in the NVE system, using a Nosé propagator^31^ with a time step of 1.5 fs (see the details in the results section) for a total number of 5000 time steps as the production job. The initial velocities are assigned randomly by SIESTA. We use the relaxed structure generated as explained above and then run a 1000 steps Nosé thermostat (at T = 300 K, 350 K etc) to produce the initial set of data for velocities and positions.

### Geometric models

**Fig. 1:**
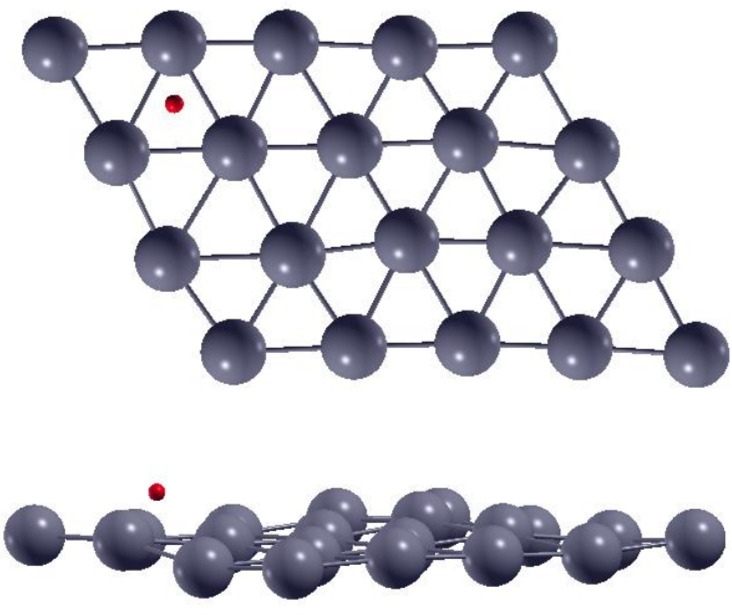
Geometric structures at T = 300 K used in the calculations. Top/bottom: top and lateral views of the topmost layers of the Zn(002) surface with an H atom on top at the initial time step of the MD calculation.

The geometric structure was generated by creating a slab model from the bulk periodic structure. The lateral size of the slab’s supercell was 4 × 5 units, while the number of layers was set to six for a total number of 120 Zn atoms in the slab. A vacuum layer of 20 Å was used to separate periodically repeated slabs along the axis perpendicular to the surface (OZ axis in our setup). The graphic representation of the structures after thermalization at T = 300 K is given in Fig. 1. The surface orientation used for all our calculations was (002).

### Thermodynamic properties and data analysis

Surface diffusion kinetics can be described in terms of ad-atoms residing at adsorption sites on a 2D lattice; in a random-walk model they are moving between adjacent (nearest-neighbor) adsorption sites (i.e. a random jumping process between equilibrium sites)^32^. The key parameter is the jump rate that is determined by two main numerical values. First, the oscillation frequency of the hydrogen atom on top of its equilibrium position in the 2D lattice, $\omega$; second, the energetic barrier between two equilibrium positions, $E_b$. Together they determine the value for the probability of an attempt resulting in a successful jump as

1 $$\begin{aligned} \mathscr {R}(\omega ) = \omega e^{-E_b/kT} \end{aligned}$$

here $\mathscr {R} (\omega )$ is the jump or hopping rate, *T* is temperature, and *k* is the Boltzmann constant. We note that the $E_b$ must be smaller than the energy of desorption for diffusion to occur, otherwise desorption processes would dominate.

The vibrational properties can be investigated via the vibrational density of states (VDOS), representing the number of vibrational states/frequency window. In the present study, the density of vibrational states was computed as:

2 $$\begin{aligned} \mathscr {D}(\omega ) = \frac{2}{kT} \sum _{j=1}^N \sum _{\alpha =1}^3 m_j {\delta }_j^\alpha (\omega ) \end{aligned}$$

where $m_j$ is the mass of the atom with index *j* and $\alpha$ is the index of the Cartesian coordinate. The quantity ${\delta }_j^\alpha (\omega )$ (spectral density) can be computed as the square of the Fourier transform of velocities^33,34^:

3 $$\begin{aligned} {\delta }_j^\alpha (\omega )=\lim _{\tau \rightarrow \infty } \frac{1}{2\tau } | \int _{-\tau }^\tau v_j^\alpha (t) e^{-i 2\pi \omega t} dt |^2 \end{aligned}$$

Finally, the diffusion coefficient, *D*, in a random-walk model is calculated as^35^:

4 $$\begin{aligned} 4Dt = \langle \Delta x^2 \rangle + \langle \Delta y^2 \rangle \end{aligned}$$

which is the two-dimensional Einstein relation, with *t* being the diffusion interval, $\langle \Delta x^2 \rangle$ and $\langle \Delta y^2 \rangle$ being the variance of 2D coordinates - directly available from the molecular dynamics data.

## Results and discussion

### Geometric properties

#### Single hydrogen atom/supercell

In order to investigate the position of the energy barrier(s) on the surface we analyze the trajectories of the hydrogen atom during the dynamics. Two temperatures were used for the propagation, i.e. T = 300 K and T = 350 K. This provides two categories of information: localization of the energy barrier and a qualitative estimation of the hopping rate between neighboring equilibrium sites.

We can see directly from the trajectory plots (see Figs. 3, 4 that the bridge position (i.e. equal distance between two Zn atoms in the surface) is the preferred jump point (i.e. it has the lowest energy barrier). We note that this is in agreement with the result in^1^ indicating that this position is energetically favorable for hydrogen.

**Fig. 2:**
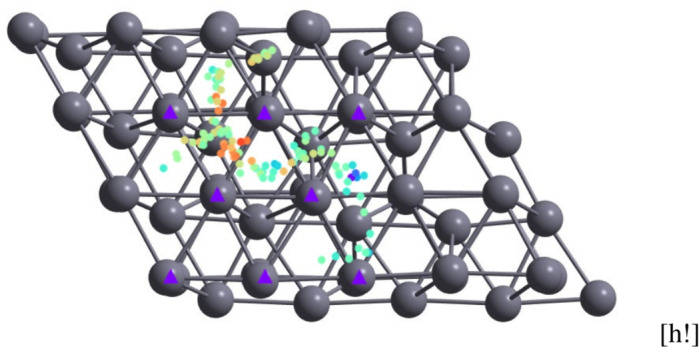
Representation of the trajectory of the hydrogen atom on top of the surface; every 30th step is used to extract the X and Y coordinates on top of the Zn(002) surface; a colored circle is plotted at the corresponding X,Y point. The color is associated with the height of the atom with respect to the surface. For the sake of clarity, this is not shown here, but is present in all schematic representations of trajectories in the figures below. Purple triangles represent the topmost Zn atoms on the surface.

In order to get an intuitive perspective on the surface dynamics of the H atom, we use the following steps. We start by recording the list of the structures of the surface-atom system for each step of the dynamics as a list of coordinates (XYZ file). To represent this in a 2D plot, we use a combination of position-color data: the actual position in the XOY plane (i.e. parallel to the surface) is represented by its Cartesian position while the height (i.e. the Z coordinate of the H atom) is represented as a color code associated with the dot at X,Y coordinates. Finally, since the number of time steps in the dynamics is too large, we select every N-th step for the representation (i.e. a dynamics with a time step N times larger) which provides an intuitive perspective on the dynamics. This method was used to analyze the data generated for different configurations and temperatures; it is summarized in Fig. 2.

**Fig. 3:**
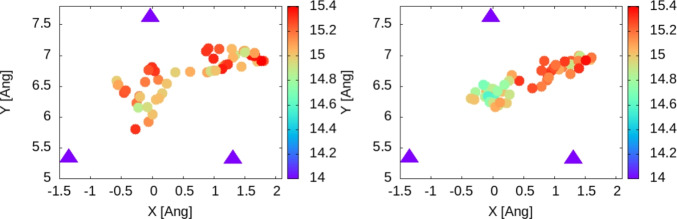
Trajectory over the first 250 steps (left), 250–500 (right). We represent only the 5-th step for the sake of clarity by extracting the X and Y coordinates of the hydrogen atom at the corresponding time steps, i.e. 1,6,11,16 ... 251 for the left plot and 251, 256, 261.... 5001 for the right and plotting them as colored circles. Triangles represent the Zn atoms in the surface. The Z coordinate of the hydrogen atom is indicated by the color of the circles and the corresponding color-bars. The average Z coordinate of the Zn atoms on the surface is 14 Å.

**Fig. 4:**
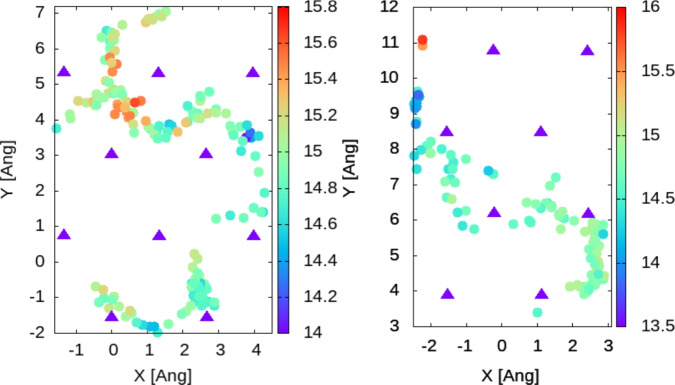
Full trajectory of hydrogen in 2D coordinates; for the sake of clarity every 30-th step is represented. By extracting the X and Y coordinates of the hydrogen atom at the corresponding time steps, i.e. 1,31,61.... and plotting them as colored circles. Triangles represent the Zn atoms in the surface. The Z coordinate of the hydrogen atom is indicated by the color of the circles and the corresponding color bars. The average Z coordinate of the Zn atoms in the surface is 14 Å. Left: data for dynamics at temperature T = 300 K; Right: data for dynamics at temperature T = 350 K.

By inspecting the trajectories in Figs. 3 and 4 we can estimate the time spent by the hydrogen atom inside each of the hollow regions (i.e. equilibrium ones). This is done directly by counting the points inside specific areas of the surface. While this is a qualitative method, let us remind that the process itself is a statistical process and the estimations of hopping rate should be based on statistical data.

By counting the number of trajectory points in Figs. 3 and 4 and multiplying with the step used in the graphical representation, we estimate a number of 300–400 steps for each hollow-like zone. This provides a first estimation for the period of the jump rate on the surface, a time of 0.45–0.6 ps, or a frequency of the jump in the range 1.7–2.2 THz.

**Fig. 5:**
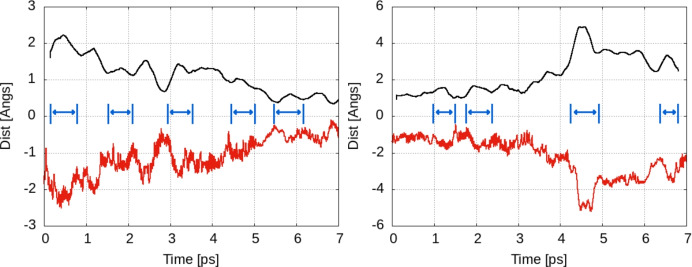
Distance between a fixed point in space (i.e. the origin) and the hydrogen atom. Black lines: window-averaged values using a time window of $\approx$ 0.2 ps. Red lines: raw data over the entire dynamics calculations (i.e. for the 5000 time steps). Left: temperature T = 300 K; Right: temperature T = 350 K. In blue we indicate some of the peaks representing oscillations of the hydrogen atom around specific positions on the surface. The jumps between these positions (i.e. hollow positions of the surface) lead to the formation of new peaks. The broadening of the peak is correlated to the jump frequency.

In order to provide a more quantitative value for this first estimation, we analyze the distance between a fixed point in space (e.g. the origin) and the adsorbed atom during the dynamics. This value is expected to display oscillations around a specific value (i.e. the distance origin-hollow position for the adsorption) as long as the atom is pinned to some equilibrium position; a sudden increase/decrease of the origin-atom distance should be observed if a jump between another position occurs. The broadening of the peaks resulting from the representation of such distance should be proportional to the time spent by the atom between two jumps. In Fig. 5 we plot these for the raw data as well as for a window-averaged distances over 25 steps. The qualitative analysis of the broadening for the peaks confirms the values around 0.4–0.6 ps for the time between jumps obtained by direct count of trajectory points, with the corresponding values of 1.6–2.5 THz.

#### Two hydrogen atoms/supercell

**Fig. 6:**
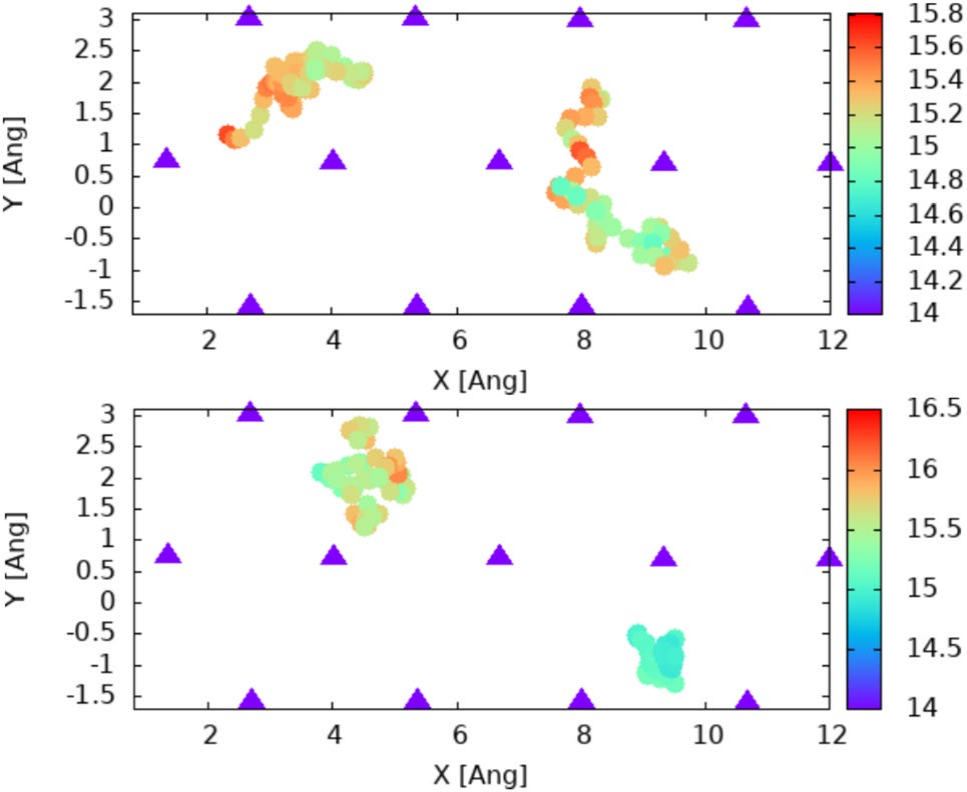
Trajectory over the first 200 steps (top) and 200–400 (bottom). We represent only the 5-th step for the sake of clarity, as explained in the caption of Fig. 3. Triangles represent the Zn atoms in the surface. The Z coordinate of the hydrogen atom is indicated by the color of the circles and the corresponding color-bars. The average Z coordinate of the Zn atoms in the surface is 14 Å .

**Fig. 7:**
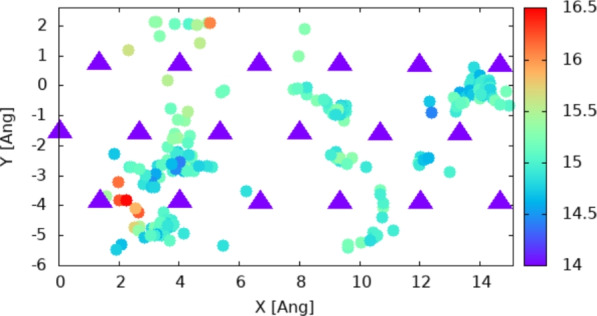
Full trajectory on the surface as 2D coordinates represented as circles; atoms in the surface are represented as triangles. Every 30-th step is represented in the plot, as explained in the caption of Fig. 4. The Z coordinate of the hydrogen atom is indicated by the color of the circles and the corresponding color-bars. The average Z coordinate of the Zn atoms in the surface is 14 Å.

We perform the same calculations as above for a model including two hydrogen atoms on the top of the slab (i.e. 4 $\times$ 5 Zn atoms in the supercell). In this case, the hypothesis of the low coverage is still valid, due to the large size of the supercell. On the other hand, the effect of hydrogen-hydrogen interaction on the diffusion process can be seen and discussed. The trajectory analysis leads to similar conclusions for the position of the transition point between sites (i.e. the bridge position), see Figs. 6 and 7.

**Fig. 8:**
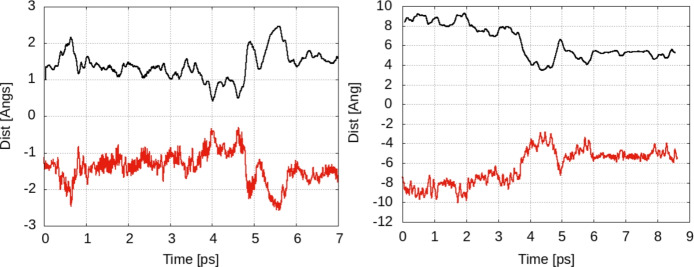
Left: Data similar to those in Fig. 5 for a model with two hydrogen atoms/super cell: the distance between the first atom and the origin is monitored to spot the change in the average, i.e. the shift from one equilibrium position to another. Right: Interatomic distance between hydrogens on the surface. Plateaus at distances around 6/8 Å can be seen. The smallest distance H–H is around 3Å but no trend for H–H forming a molecule can be seen. Red lines: raw data for all MD steps; black lines: window average for a time step of 0.2 ps.

The qualitative estimation of the hopping rate for a 2H model was done using the analysis of the distance between the atom and the origin. Data are given in Fig. 8 and indicate an increasing trend for the jump rate; while still very qualitative, the broadening of the peaks is shifted toward 0.3–0.5 ps (i.e. 2 to 3 THz).

In addition to the trajectory analysis for a single atom, in the two-hydrogen atoms model we can plot the time dependence of the distance between the two hydrogen atoms (see Fig. 8). The distance monitors the possible presence/formation of a hydrogen molecule on the surface (i.e. H–H distance around 0.8 Å ). We note that the minimum distance reached in Fig. 8 is around 3 Å . This value is still far from the bond length of the hydrogen; we do not see any trend to trigger a dynamics encouraging the formation of the molecules on the surface. Indeed, the distance is kept for a short period of time (around 1 ps) then a trend of keeping a constant distance of 6–8 Å is present for the whole evolution of the system. Such distance corresponds to a maximization of the distance between the atoms in the periodic boundary conditions slab model with a lateral size around 15 Å . We conclude that in the low coverage model the systems seem more stable at H–H interatomic distances that are not favorable for the formation of molecular hydrogen or even encourage the repulsion between hydrogens.

### Vibrational density of states

#### Single hydrogen atom/supercell

The vibrational density of states of a hydrogen atom’s vibrations on top of a zinc surface is essential for the understanding of the Eq. 1.

**Fig. 9:**
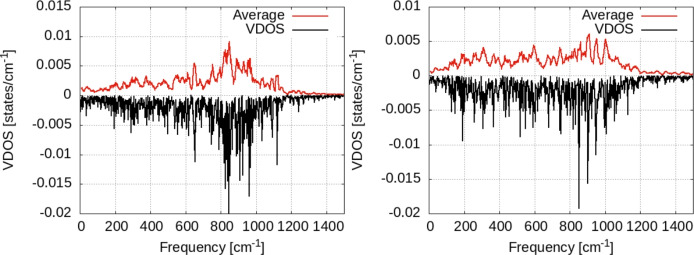
Vibrational density of states for the hydrogen atom adsorbed on top of the Zn surface. Black lines: raw data; red lines: window-averaged data over 5 frequencies. Temperature of the systems: T = 300K (left), T = 350K (right).

**Fig. 10:**
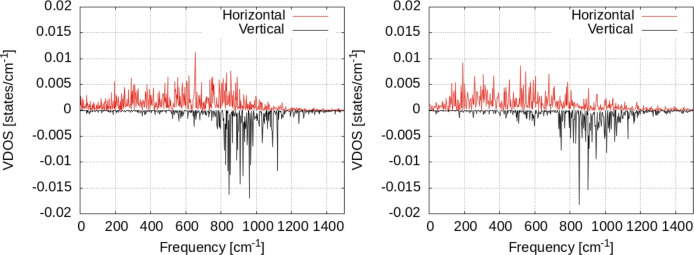
Vibrational density of states for the hydrogen atom adsorbed on top of the Zn surface, separated over horizontal/vertical vibrational modes with respect to the surface. Temperature of the systems: T = 300K (left), T = 350K (right).

**Fig. 11:**
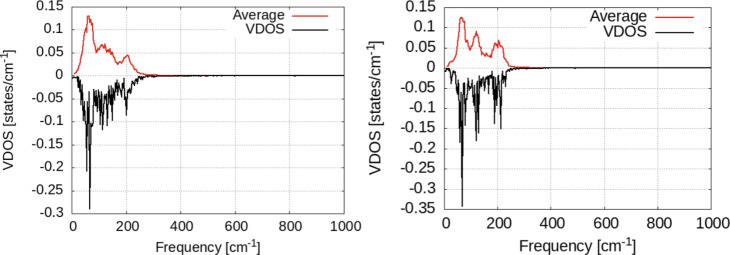
Vibrational density of states for the Zn atoms in the surface. Black lines: raw data; red lines: window-averaged data over 5 frequencies. Temperature of the systems: T = 300K (left), T = 350K (right).

Our results are presented in Fig. 9 for 5000 steps of Nosé dynamics with the hydrogen atom on the surface at T = 300 and 350 K. The VDOS for a single hydrogen atom shows the differences between the two as an effect of temperature increase. Most important of them is the increase of the VDOS at low frequencies for T = 350 K. Also, the whole spectrum is more uniformly distributed over the whole frequency range, as temperature increases. Interestingly, we see that the distribution of the VDOS in the surface and perpendicular to the surface is not homogeneous. The data in Fig. 10 were obtained by summing the values in the Fourier transform in Eq. 2 over the horizontal (i.e. in the plane XOY ) and transversal (i.e. parallel to OZ axis). Almost the entire VDOS at low frequencies (i.e. below 400 cm^−1^) is transversal VDOS; this is the result of vibrational coupling between the vibrational motion of the hydrogen to the Zn surface (see the VDOS of the Zn surfaces in Fig. 11 below). Moreover, the temperature increase of the VDOS is also caused by this motion; finally, the VDOS at zero frequency is responsible for the diffusion on the surface^36^. It can be seen that only the in-plane components are responsible for this. Let us also note that the effect of temperature on the VDOS of the Zn atoms in the surface can be seen in Fig. 11. Here too, the increase in temperatures leads to populating vibrational energy states more uniformly over the entire spectrum of frequency (i.e. 0–200 cm^−1^).

**Fig. 12:**
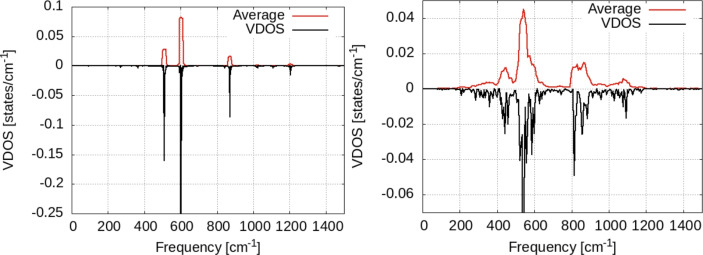
Vibrational density of states for the hydrogen atom adsorbed on a frozen surface (Zn atoms are pinned to their positions) for a single H atom (left) and two atoms. Black lines: raw data; red lines: window-averaged data over 5 frequencies. Left: data for T = 300 K; right: data for T = 350 K.

In order to spot the effect of vibrational coupling between hydrogen and the surface, we calculate the VDOS for the H adsorbed on a frozen surface, i.e. the hydrogen atom vibrates on top of fixed Zn atoms. We see that for the hydrogen on top of the frozen surface (Fig. 12) no vibrational density of states is present at zero frequency-suggesting that in this case no diffusion takes place on the surface^36^. The entire spectrum of VDOS in this case consists of three narrow bands at 510, 615 and 900 cm^−1^. The first two are in-plane (horizontal) vibrations while the third one is perpendicular to the surface (see Fig. 12).

By comparing this to the data discussed above (i.e. the motion of hydrogen is coupled to the vibrations of Zn atoms in the surface), we see that coupling to the surface leads to new and/or broader bands that are present at 1000 and 1100 cm^−1^ with no correspondence in the spectrum of hydrogen on the frozen surface.

**Fig. 13:**
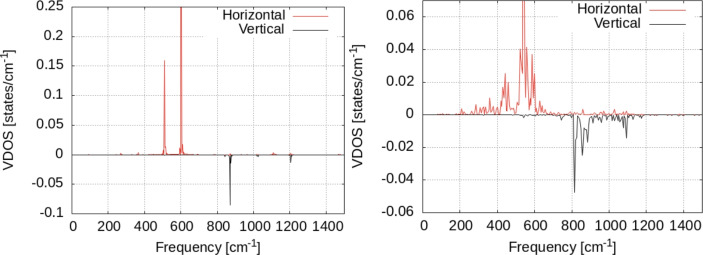
Vibrational density of states for the hydrogen atom adsorbed on a frozen surface, separated over horizontal/vertical vibrational modes with respect to the surface.

The presence of the second hydrogen atom on top of the frozen surface leads also to a significant broadening of the VDOS bands, as seen in Fig. 12. Indeed, the larger frequencies (i.e. above 1000 cm^−1^) can be assigned to the H–H interaction (probably indirect, via substrate). However, no VDOS is present at zero frequency in this case too. Consequently, it can be argued that the vibrational coupling between hydrogen and the substrate plays a central role (if not the central) in the hydrogen migration on the Zn surface (see the Fig. 13).

#### Two hydrogen atoms/supercell

**Fig. 14:**
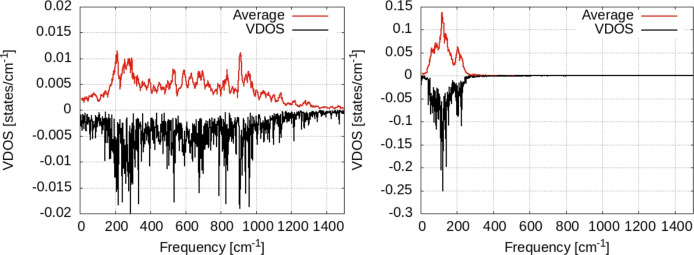
Vibrational density of states for the atoms on the surface. Left: density of states for the two hydrogen atoms. Right: density of states for the Zn atom in interaction with hydrogen. Black lines: raw data; red lines: window-averaged data over 5 frequencies.

**Fig. 15:**
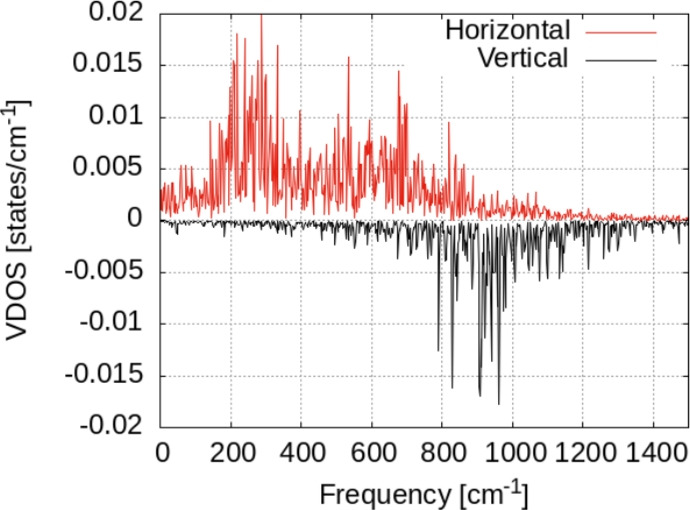
Vibrational density of states for the hydrogen atoms adsorbed on the surface, separated over horizontal/vertical vibrational modes with respect to the surface.

The vibrational density of states for the geometric model with two hydrogen atoms on top of the supercell slab is presented in Fig. 14. We note that the conclusions from the discussion of VDOS for a single hydrogen atom are qualitatively the same in this case. The peaks around 900, 600 and 300 cm^−1^ are present in this case too. The VDOS at 200 cm^−1^ becomes dominant in this case. According to previous discussions, this is probably the results of H–H interaction and the vibrational coupling with the surface. Indeed, the VDOS of the Zn surface alone displays a maximum of VDOS around 130 cm^−1^, as seen in Fig. 15. A smaller intensity is noticed for the peaks above 1000 cm^−1^. On the other hand, at low frequencies the VDOS is clearly non-zero indicating the presence of very low frequencies of oscillation, and translation at frequency zero.

By projecting the VDOS on vertical-horizontal components, we see clearly that the horizontal part becomes dominant, most probably as a consequence of the H–H interactions. While this interaction does not lead to the formation of a molecule (i.e. attractive interaction between atoms), it can transfer an important energy to the lateral oscillations of the atoms. The effect of this on the diffusion probability is difficult to estimate; since the effect of increasing probability by increasing the VDOS is somehow compensated by the effect of lowering the vibrational frequency of the VDOS (i.e. around 200–300 cm^−1^).

### Diffusion coefficients

This section is dedicated to the calculation of the diffusion coefficient on the surface based on the molecular dynamics data. The simple equation for the jump rate given in Eq. 1 describes the dependence of the jump probability on a single vibrational frequency, $\omega$. Since the vibration of adsorbed hydrogen involves a whole spectrum of vibrational frequencies, i.e. VDOS, $\mathscr {D} (\omega )$, Eq. 1 should be re-written as an integral over the independent probabilities at each frequency, i.e.

5 $$\begin{aligned} \mathscr {R} = \int \mathscr {D} (\omega ) \omega e^{-E_b (\omega ) /kT} d \omega \end{aligned}$$

where we assume that the energy barrier has also some dependence on the vibrational mode, as a consequence of the deformation occurring on the surface. In other words, the shift of the vibrational frequency in the VDOS is reflected in a shift of the hopping rate on the surface. This model allows us to give a qualitative comment on the hopping rate as function of the coverage and temperature, as a result of the data in Figs. 9, 10, 11, 12, 13 and 14.

The jump frequency was estimated above to be around 2 THz i.e. 66 cm^−1^; for vibrational frequencies around 600 cm^−1^ (i.e. as in the frozen surface model) we get the values of the exponential term in Eq. 1 to be around 0.1. This leads to an energy barrier of 2–3 kT, which is 0.08–0.06 eV.

**Fig. 16:**
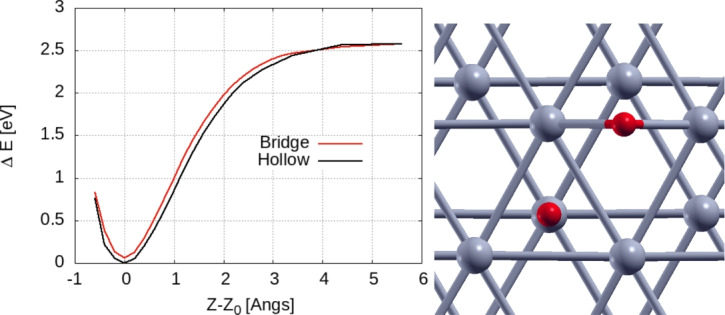
Left: Energy curve, *E*(*Z*), *Z* = atom surface distance for the interaction of a hydrogen atom with the Zn surface. Two initial (minimum energy) positions are taken into account: hydrogen in the hollow position (stable position) and hydrogen in the bridge position (i.e. transition state for the diffusion process). Right: geometric representation of the two positions used in calculations (i.e. bridge and hollow).

In order to check the validity of this estimation, we calculate explicitly the energy barrier between the hollow and bridge position by relaxing the atomic positions in the entire system and allowing hydrogen to move only along the OZ axis for the saddle position. Figure 16 indicates the energy dependence of the total system on the displacement of the H atom along the OZ axis. In each case, the origin was set to the point where the hydrogen reaches its equilibrium (i.e. the lowest energy for the entire system). It can be seen that an energy barrier does exist between the two systems, with a similar total energy at “infinite separation” between the surface and atom. The calculated value of the gap (i.e. the difference between the two lowest energies) is 0.057 eV-in full agreement with the qualitative data obtained from the diffusion data above.

**Fig. 17:**
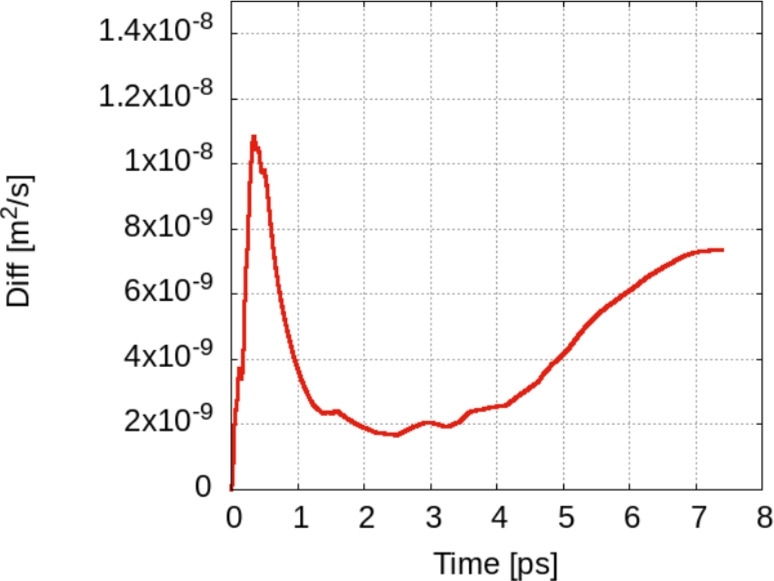
Convergence of the diffusion coefficient over time: $D=(\langle \Delta x^2 \rangle + \langle \Delta y^2 \rangle )/4t$. The limit value is $\approx 7.5 \times 10^{-9}$. The experimental value at $\hbox {T=50}^{\circ }$ Celsius is around $6.5 \times 10^{-8}\;\hbox {m}^2/{\text{s}}$^37^.

The calculated diffusion rates for low-coverage models used in our models according to Eq. 4 are around $10^{-8}\;\hbox {m}^2/s$, at 300 K (see Fig. 17), which is about 4 times less than the experimental values^37^. Several comments have to be made in order to compare this with experimental data, we have to take into account two specific features of the model used in the calculation.

First, we use a model with a perfect flat surface (no defects, kinks, terraces etc) and a low coverage regime (i.e. single hydrogen atoms adsorbed for several atomic sites on the Zn surface. This model has the tendency to bind stronger the hydrogen atom to specific sites which will decrease the diffusion coefficient. Defects on the surface tend to increase the mobility since the interaction between hydrogen and an irregular surface is weaker than that on perfect ones. On the other hand, the presence of other hydrogen atoms has also the tendency to increase the hopping probability as is already visible on the VDOs plots of two atoms, as compared to those with a single atom.

Second, the experimental data in^37^ are data for the bulk. Some of the comments above-like the effect of neighbors and defects still apply in this case. All these allow us to conclude that the experimental rate should be larger than that calculated in our ideal-low coverage model. We cannot indicate a factor, but the value of 4 is reasonable if we take into account all the comments above.

The list of calculated diffusion coefficients for all models (single hydrogen at 300/350 K, two hydrogens at 300 K and the frozen surface) is given in Table 1. We see the dependence on temperature (about 10% increase between hydrogen at 300 K and at 350 K). Also, we note the increase in the coefficient for two hydrogens/unit cell on the surface. This is in qualitative agreement with our data indicating an increase of jump probability toward 3 THz as well as a repulsive H–H trend we noticed in the H–H distance plots. Finally, the values for the frozen surface is three orders of magnitude than those for models including surface phonons.

Let us note that the impact of surface phonons on hydrogen diffusion, as emphasized by our results, is twofold: a direct effect, consisting of the energy coupling between the vibrational modes parallel to the surface and hydrogen (see the range 0–200 cm^−1^ in the VDOS of the surface versus that of adsorbed hydrogen). In this case, additional kinetic energy becomes available for the hydrogen to overcome the barrier. The second effect is more indirect and is related to binding/potential energy of the hydrogen on top of the surface. Indeed, by inspecting Fig. 16 for the energy of the H atom on top of Zn, it can be argued that any deformation of the Zn surface brought by the vibrational motion will result in a shift/change of the energy curves as a result of the different orbital overlap (i.e. an essentially geometric parameter) between Zn and H. This will change dynamically the potential energy landscape, probably lowering the barriers since the data in Fig. 16 represent the values for the lower boundaries of the Hamiltonian. The molecular dynamics data represent an average over all these effects.

**Table 1:** Diffusion coefficient estimated using Eq. 4 for different models: single hydrogen atom/supercell at 300 K, similar at 350 K, two hydrogen atoms/supercell at 300 K and a hydrogen atom on top of a frozen Zn surface.

Model	1H/300K	1H/350K	2H/300K	1H/Frozen
$D [10^{-8} \;{\text{m}}^2/{\text{s}}]$	0.75	1.3	1.2	$10^{-4}$

Hydrogen diffusion on the zinc surface enables and accelerates the hydrogen evolution reaction (HER), which worsens zinc corrosion, reduces battery efficiency, and shortens the life of zinc–air batteries. Controlling this diffusion is essential to improving the performance and durability of zinc–air systems. Our results clearly show that one of the strategies to follow in order to reach this goal has to be related to the mechanisms that control or reduce the vibrational energy of the surface, like surface coatings that can change the phonon spectra of zinc.

## Conclusions

We studied hydrogen diffusion on zinc surfaces in the low-coverage regime, where hydrogen atoms predominantly jump between vacant sites due to the low probability of collisions with other hydrogen atoms. Using theoretical approaches, including Density Functional Theory (DFT) and molecular dynamics simulations, we analyzed the dynamic processes governing hydrogen diffusion. Additionally, we assessed the accuracy of these methods by comparing our results with experimental data.

We found typical values for the jump frequency between two sites of the Zn (002) surface between 1.5 and 2.5 THz and an energy barrier for the diffusion with 0.06 eV high. These values are confirmed by molecular dynamics as well as by direct calculation of the DFT corresponding energies. The calculation of vibrational modes for adsorbed hydrogen reveals that the vibrational coupling between the horizontal modes of phonons in the surface and the hydrogen is essential for the diffusion to occur. Our calculation shows that the diffusion coefficient is close to $10^{-8}\;{\text{m}}^2/{\text{s}}$ at room temperature, which is qualitatively in agreement with the experimental results. On the other hand, hydrogen on top of a frozen surface has a diffusion coefficient about $10^{-4}$ smaller compared to the realistic case (i.e. vibrating surface)—i.e. practically no diffusion is present if the atoms in the metallic surface do not vibrate.

We note that our results are obtained in the low coverage regime for a perfect surface; for a better understanding of the full mechanism of hydrogen diffusion calculations at high coverage are necessary. As a first step, we performed calculations with two atom/unit cell; while still in the low coverage regime, the interaction between two hydrogen atoms leads to an increase in the diffusion rate; remarkably, this increase is also correlated with significant changes in the vibrational density of states of the surface.

Therefore, while additional studies are needed to understand the behavior of hydrogen on fully covered surfaces, based on current data it can be suggested that hydrogen diffusion on the surface can be partially regulated by designing surfaces with a low vibrational density of states while maintaining the chemical composition. This can be achieved through various techniques that create nanostructures on the surface^38^, acting as phonon traps. These structures may serve as diffusion inhibitors, ultimately controlling the hydrogen evolution reaction on zinc and enhancing the practical performance of zinc–air batteries.

## Data Availability

The datasets used and analysed during the current study are available from the corresponding author on reasonable request.
